# Wearable Health Technology and Electronic Health Record Integration: Scoping Review and Future Directions

**DOI:** 10.2196/12861

**Published:** 2019-09-11

**Authors:** Catherine Dinh-Le, Rachel Chuang, Sara Chokshi, Devin Mann

**Affiliations:** 1 Department of Population Health New York University School of Medicine New York, NY United States; 2 Accenture New York, NY United States

**Keywords:** wearable electronic devices, electronic health records, data collection, mobile health, patient monitoring

## Abstract

**Background:**

Due to the adoption of electronic health records (EHRs) and legislation on meaningful use in recent decades, health systems are increasingly interdependent on EHR capabilities, offerings, and innovations to better capture patient data. A novel capability offered by health systems encompasses the integration between EHRs and wearable health technology. Although wearables have the potential to transform patient care, issues such as concerns with patient privacy, system interoperability, and patient data overload pose a challenge to the adoption of wearables by providers.

**Objective:**

This study aimed to review the landscape of wearable health technology and data integration to provider EHRs, specifically Epic, because of its prevalence among health systems. The objectives of the study were to (1) identify the current innovations and new directions in the field across start-ups, health systems, and insurance companies and (2) understand the associated challenges to inform future wearable health technology projects at other health organizations.

**Methods:**

We used a scoping process to survey existing efforts through Epic’s Web-based hub and discussion forum, UserWeb, and on the general Web, PubMed, and Google Scholar. We contacted Epic, because of their position as the largest commercial EHR system, for information on published client work in the integration of patient-collected data. Results from our searches had to meet criteria such as publication date and matching relevant search terms.

**Results:**

Numerous health institutions have started to integrate device data into patient portals. We identified the following 10 start-up organizations that have developed, or are in the process of developing, technology to enhance wearable health technology and enable EHR integration for health systems: Overlap, Royal Philips, Vivify Health, Validic, Doximity Dialer, Xealth, Redox, Conversa, Human API, and Glooko. We reported sample start-up partnerships with a total of 16 health systems in addressing challenges of the meaningful use of device data and streamlining provider workflows. We also found 4 insurance companies that encourage the growth and uptake of wearables through health tracking and incentive programs: Oscar Health, United Healthcare, Humana, and John Hancock.

**Conclusions:**

The future design and development of digital technology in this space will rely on continued analysis of best practices, pain points, and potential solutions to mitigate existing challenges. Although this study does not provide a full comprehensive catalog of all wearable health technology initiatives, it is representative of trends and implications for the integration of patient data into the EHR. Our work serves as an initial foundation to provide resources on implementation and workflows around wearable health technology for organizations across the health care industry.

## Introduction

### Electronic Health Record Adoption and Expanded Access to Patient-Collected Data

Although electronic health records (EHRs) date back to the 1960s, widespread adoption was stagnant until the more recent passage of the Health Information Technology for Economic and Clinical Health Act in 2009 [1-5]. Between 2001 and 2011, the number of physicians using EHR systems increased from 18% to 57% [6]. Policies such as Meaningful Use (which prioritized quality, care coordination, and security of personal health information) incentivized the continued adoption of EHRs. By 2015, nearly 9 in 10 (87%) of office-based physicians adopted an EHR system [7-9]. Of all EHR vendors, Epic, Cerner, and Meditech are the most prevalent among health care systems [10].

In addition to driving EHR adoption among providers and health systems, legislation supporting meaningful use also paved the way for continued development of EHR capabilities to enhance the patient experience. Health systems are increasingly interdependent on EHR capabilities, offerings, and innovations to better capture patient data [11]. Features include secure messaging with patients and features to view, download, and transmit their EHR. Such capabilities are becoming more prevalent to facilitate streamlined patient data exchanges with their provider [7].

A novel capability offered by health systems encompasses the integration between EHRs and medical devices, including wearable health and fitness tracking devices. Although early device integration involved tracking a set of simple vital signs, the scope of patient data has expanded rapidly as health systems strive to meet new standards, new care models, as well as leverage innovation in digital technologies [12,13]. The primary focus of this review was to capture a sample of the rapidly changing field of patient data integration into the EHR [14]. Specifically, we review several health systems and organizations that are using patient data gathered through consumer-grade wearable devices to track and improve patient outcomes.

#### Availability and Adoption of Wearable Devices

Wearable devices include wristbands, smartwatches, wearable mobile sensors, and other mobile *hub* medical devices that collect a large range of data from blood sugar and exercise routines to sleep and mood. Patient data are collected either through consumer reporting or passively through sensors in apps that communicate with devices through application programming interfaces (APIs); these data are then shared through data aggregators such as Apple’s HealthKit that pools data from multiple health apps [15].

**Figure 1:**
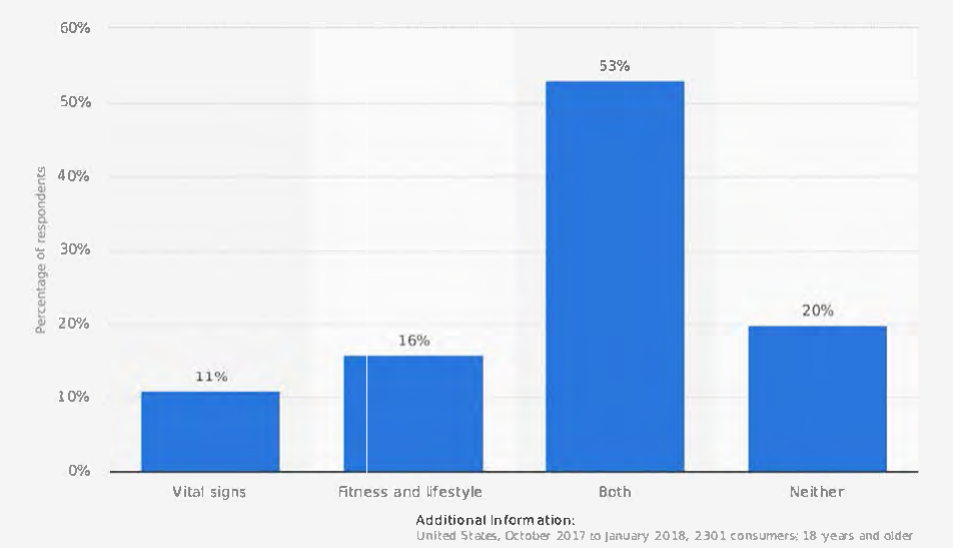
Percentage of US adults who were willing to wear technology that tracks select health statistics as of 2018. Screenshot from www.statista.com [16].

According to a recent consumer survey on digital health by Accenture, a significant percentage of US adults were willing to wear technology that tracks their health statistics (see Figure 1) [16]. Due to mobile integration platforms such as Google Fit and Apple HealthKit, we can expect to see an increase in the number of health-wearable users over the next few years [17,18]. The upward trend in device usage to monitor health-related data additionally suggests there will be a correlated rise in patient data available for health management [19]. Large health systems are likely to trend toward larger rollouts of wearable technology in the next few years, potentially incorporating wearables as part of their preventative care strategy by monitoring heart rate, blood pressure, and other information [20,21]. There are currently more than 400 EHR-compatible devices on the market, a number that is expected to rise exponentially in the coming years [22].

#### Clinical Impact of Wearable Devices

Currently, these devices have the potential to help patients and providers manage chronic conditions such as diabetes, heart conditions, and chronic pain [23-25]. According to the Pew Research Center, 60% of US adults reported tracking their weight, diet, or exercise routine; 33% of US adults track health symptoms or indicators such as blood pressure, blood sugar, or sleep patterns; and 8% of adults specifically use medical devices, such as glucose meters [26]. Studies on the clinical impact of wearables on patient health outcomes offer varied results. Although some conditions such as physical activity and sleep did not show significant or conclusive change from wearable technology use and require further evaluation, other studies have reported improved subjective outcomes on patient health [27,28].

Recent literature reviews on the clinical impact of wearable devices and behavior change have shown promising effectiveness for digital technology [29]. However, much of the literature calls for more complete data analyses from commercially available tools and their impact on patients [30,31]. Further studies are necessary to assess clearer clinical outcomes on patient health by wearable health technology.

The purpose of this paper was to conduct a scoping review of the wearable health technology field to provide an overview of current wearable innovations in the EHR. Similar to a number of existing scoping reviews, we used internet search engines in addition to our database searches to capture the rapid updates in the area of health system integration of remotely collected patient data [32]. We used these sources to generate a targeted list of organizations that are leaders in the overall field of wearable health technology, along with their partnerships.

This paper provides an overview of (1) our process in determining the current landscape of wearable health technology and (2) descriptions of some leading innovations and partnerships by start-ups, providers, and insurance companies. By sharing our results, we hope to create a process to identify relevant organizations in this field and provide resources for organizations that are interested in joining or learning more about implementation and workflows around wearable health technology and patient data integration to EHRs. This study is specific to integration into the Epic portal and is not a comprehensive search; however, results are representative of the field because of Epic’s prominence in the US acute care hospital market (25.8%) [10].

## Methods

### Search Process

To better understand the scope of wearables and other health tracking devices and the resulting impact on EHRs, we used a scoping process to survey existing efforts on the Web. Although not directly relevant to a scoping review, we reviewed Preferred Reporting Items for Systematic Reviews and Meta-Analyses guidelines to enhance the quality of our search. In our search, to identify the leaders in the field, we contacted the largest commercial EHR system (Epic) for information on client work in the integration of patient-collected data. We used this information to further inform our search terms in Epic’s UserWeb portal, the primary platform for Epic users to share and discuss topics such as innovative idea generation and event postings, and the general Web, PubMed, and Google Scholar database searches from July 2018 to January 2019.

We recognize the risk of bias in this study, as our search process was limited to Epic clients and the information publicly available on the World Wide Web.

### Inclusion Criteria

We used a set of inclusion criteria in UserWeb to ensure that postings were accurate and up to date. Results had to meet the following standards: (1) be posted after June 2017 and (2) have responses to topic threads. Key search terms included Apple HealthKit, Patient remote data integration, Fitbit integration, Withings integration, and Wearables.

Similarly, our findings on wearable technology companies and initiatives from the general Web, PubMed, and Google Scholar database searches had to meet the following criteria: (1) be posted after June 2017 and (2) match search terms including but not limited to Device integration, EHR data integration, Epic MyChart integration, Patient MyChart integration, Patient remote data integration, Patient data access, Wearables, Provider wearables, Hospital wearables, Hospitals AND Apple HealthKit device integration, Apple HealthKit device integration AND Epic, Start-ups AND EHR integration, Insurance companies AND device integration, APIs AND device integration.

## Results

### Challenges of Wearable Device Integration

Although wearable health technology has the potential to transform patient care, issues such as concerns with patient privacy, system interoperability, and the immense amount of patient data pose a challenge to the adoption of wearables by providers [33,34]. Such challenges are critical to consider for future wearable use to deliver safe and quality care for patients. Although there are potential solutions for these implementation issues, more innovative work is required for wide-scale adoption of wearable health technology.

#### Protecting the Confidentiality and Privacy of Patients

Wearable health technology requires critical checkpoints along the workflow to protect the confidentiality and privacy of patients [35]. Currently, there is limited empirical evidence in the literature on the appropriate implementation of security in wearable devices [36,37]. Key considerations include Health Insurance Portability and Accountability Act of 1996 (HIPAA) compliance and informed consent by wearable users.

**Figure 2:**
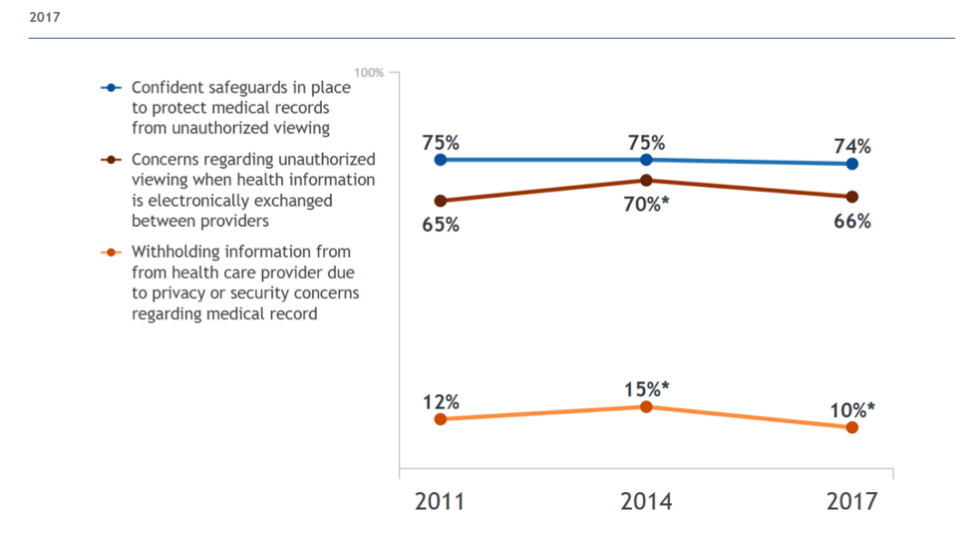
Individuals’ perceptions of the privacy and security of medical records and health information exchange in 2017. Screenshot from https://dashboard.healthit.gov/quickstats/quickstats.php [7].

The HIPAA is a US legislation that protects the privacy of individuals’ medical records and applies to health providers and plans [38]. With the continuous stream of data from personal devices, data privacy and security for health information must be addressed as to meet HIPAA standards and not impede patients’ willingness to share their data [39]. Figure 2 demonstrates that patients have some concerns about the electronic exchange of data between providers; the percentage of individuals expressing these concerns has remained relatively the same since 2011 [7]. To protect against potential cybersecurity attacks and missing or stolen patient records through the implementation of wearable health technologies, hospitals must ensure that devices are connected to a secure network and monitor the hospital data network continuously [40]. To prioritize data privacy, health systems are likely to be required to set up another secure network for wearable devices, separate from the main network [41].

The complexities of wearables continue to grow as patient datasets from wearable devices are compiled and transferred [42]. Obtaining patient consent is also critical, as patients are likely to find constant physiological surveillance to be intrusive [43]. Misuse of personal health information by third parties could lead to discrimination, changes in insurance coverage, or even identity theft [15]. As a result, consent notices must provide enough detail regarding what and how often personal information is collected and specify the third parties that can access patient data, ensuring that informed consent by the patient occurs [42,44]. Additional policies and standards are necessary for the future of wearable health technology and patient data integration to the EHR to ensure the confidentiality and privacy of patients.

#### Lack of System Interoperability and Connectivity

As the integration of patient data through wearable devices is a relatively new area of health technology, health systems are lacking the necessary platforms to pull continuous streams of data from different patient devices for integration into the EHR [45]. Currently, device and EHR vendors use a range of methods that include distinct, proprietary, and closed communication methods [46,47]. These differences in methods make it difficult for various devices and EHR systems to communicate and transfer data streams, leading to the lack of system interoperability.

As a result, this barrier has created subsets of data collected from patients that become secondary in value because they cannot be easily integrated into patient historical data [48,49]. Researchers have recently looked to achieve *plug-and-play* interoperability to standardize platforms and integrate these information islands, a standard that already exists in the world of consumer electronics as consumers demand simple and seamless functionality [47]. *Plug-and-play* standards require ease of use, device compatibility, and streamlined scalability and reconfigurability between different vendors; systems must be able to detect new devices, negotiate communication, and allow devices to synchronize and work with each other [50].

As the need for system interoperability grows, third-party applications aimed to address interoperability issues have become more prominent [45]. Increased partnerships and opportunities between makers of these applications and health systems are necessary to reach high interoperability and streamlined communication between EHR platforms, patient devices, and providers. Improving these relationships can improve health care efficiency, provider safer transitions of care, and help lower health care costs [51].

#### Patient Information and Data Overload

Wearable health technology that is integrated into the EHR produces an enormous amount of data that require compilation and interpretation before becoming useful for patients and providers [43,52]. Storing daily patient data streams can be a barrier to health systems that are not prepared to host a database that is constantly growing [53]. Decisions around the life cycle of such data and how it can best fit into provider workflows pose a unique challenge to using remotely collected data for patient care [52]. For example, the Apple Health and PulseOn Android apps provide heart rate data at 60-second long and 3-second long intervals, respectively; transmission of such large volumes of data will require backend analysis to be processed into a simpler and more usable form [54].

Due to the sheer volume of these data, extracting and presenting providers with necessary patient data has been a main discussion point among hospitals implementing wearable technology. Overall, many providers experience alert fatigue in their daily clinical decision support systems [55]. Although machine learning and artificial intelligence (AI) algorithms are potential solutions to this issue, current algorithms are often tested in fixed conditions that are not likely to hold up in live scenarios [35]. Successful solutions to patient data integration should be able to sift through the immense amount of data and automatically deliver meaningful and actionable items to providers [56].

In addition, a strong user interface (UI) for providers is important for provider buy-in and engagement during implementation. As a result, there has been an increasing trend within health care organizations to incorporate user experience and UI designers into a cross-functional information technology (IT) team to address this need [57]. The multidisciplinary skills of such teams can offer improved UIs combined with IT expertise and enhance the ability to comprehend wearable patient data. These improvements in provider engagement and workflow could improve overall time efficiency for providers and quality of care for patients.

### Innovations in Wearable Health Technology

In response to these challenges, a number of health systems and organizations have begun to use a user-centered design approach to adapt workflows and collaborate with third-party applications to improve their integration of remote patient data [58,59]. Numerous health care providers have piloted and/or implemented wearable-EHR integration projects with Apple Health, Google Fit, Fitbit, Nokia, and Withings [60]. A number of devices on the market have the capability to connect directly to EHRs through HealthKit and Google Fit; simple data such as steps and weight are currently collected and displayed, with more devices and data types being brought on the Web over time [58,60]. In addition, as of October 2018, Epic customers representing at least 565 hospitals and 14,427 clinics support connecting data from Fitbit, HealthKit, or Withings today. Epic customers representing at least 1152 hospitals and 24,496 clinics support connecting other devices through Health Level-7 or manual entry of patient data through MyChart. Note that this is not a comprehensive list of all customers, as select organizations opted out of the data collected by Epic (data provided by Epic, October 2018).

However, EHRs still cannot connect to many other devices and require the development of new solutions to address challenges such as interoperability and visualization for the information they are currently collecting [61]. The wearable health technology space features numerous start-up partnerships with health care providers and insurance company innovations that are working to address these key challenges and promote growth in wearable usage and EHR integration capabilities.

The overall themes that we used to describe the different focus areas of each partnership included personalized patient experience, rewards program, data analytics, remote monitoring, access to patient records, and AI technology. A summary of key organizations working in wearable health technology compiled from the general Web search and Epic’s UserWeb portal (as of May 2018) is presented in Tables 1 and 2, respectively.

### Start-Up Partnerships

As listed in Table 1 below, we identified the following 10 start-up organizations that have developed or are in the process of developing technology to improve wearable health technology and/or patient data integration to EHRs: Overlap, Royal Philips, Vivify Health, Validic, Doximity Dialer, Xealth, Redox, Conversa, Human API, and Glooko. We reported sample start-up partnerships with a total of 16 health systems in addressing challenges of meaningful use of device data and streamlining provider workflows. The partnerships between these start-ups and health systems serve to improve the data collection process, synthesize actionable information for providers to review, and create a more personalized experience between patients and providers. Due to the rapidly moving field of wearables, our research represents a snapshot in time of wearable health technologies and is not meant to be a fully exhaustive list.

**Table 1:** Wearable health technology start-up partnerships.

Start-up organizations	Select hospital partnership(s)	Theme(s)	Technology overview
Overlap 2019 [62]	Columbia University Medical Center and UC Davis Health	Data analytics and remote monitoring	Collects patient data through a customizable Overlap app that integrates with EHRs^a^ and various wearable devices
Royal Philips 2019 [63]	New York Presbyterian	Data analytics and remote monitoring	Helps physicians monitor patient health remotely and connect with 2-way video using a telehealth platform
Vivify Health 2018 [64]	Children’s Health in Dallas and Ascension Health	Remote monitoring	Integrates patient mobile devices with EHRs through a remote care platform
Validic 2018 [65]	Kaiser Permanente and Mayo Clinic	Data analytics and remote monitoring	Simplifies collected health data from wearables and wellness applications and delivers comprehensive patient profiles to providers
Doximity Dialer 2018 [66]	Johns Hopkins Hospital	Access to patient records and personalized patient experience	Allows providers to access their patients’ records and make patient calls on the go from their personal cell phones, using the office as the caller ID^b^ while on personal phones
Xealth 2018 [67]	Providence Health & Services and University of Pittsburgh Medical Center	Personalized patient experience	Allows doctors to prescribe apps and digital tools to their patients. Doctors can also track patient’s use of these tools from the EHR
Redox 2018 [68]	Brigham and Women’s Hospital	Data analytics	Links hospitals’ EHR systems to outside applications regardless of software vendor (Epic, and Allscripts)
Conversa 2018 [69]	Northwell Health and Ochsner Health System	Artificial intelligence technology and personalized patient experience	Allows providers to monitor patient status between visits through automated, personalized patient-provider conversation experiences. Patient also can send information through Conversa into their EHRs
Human API 2018 [70]	Mount Sinai and Cedars-Sinai	Data analytics	Pulls health data in real time and processes and normalizes actionable health data, regardless of source or original format
Glooko 2019 [71]	Mayo Clinic and Novant Health	Data analytics, personalized patient experience, and remote monitoring	Provides daily insights to people with diabetes through a mobile app; clinicians are able to access data and identify high-risk patients

^a^EHR: electronic health data.

^b^ID: identification.

**Table 2:** Insurance companies.

Organization	Theme(s)	Technology overview
Oscar Health 2018 [72]	Rewards program	Uses an app that synchronizes with Apple Health for its step-tracking program. More than three-fourths (80%) of Oscar members who download the app use step tracking
United Healthcare 2018 [73]	Rewards program	Offers UnitedHealthcare Motion program where members can earn money toward out-of-pocket medical expenses by walking. The United Healthcare Motion app syncs with wearables using Qualcomm Life's 2net Platform to track steps
Humana 2018 [74]	Personalized patient experience and rewards program	Launched Go365, a wellness and rewards program for members in 2017. The program operates on a points system and incentivizes healthier behavior with personalized health assessments and rewards, such as fitness gear and electronic devices
John Hancock 2018 [75]	Rewards program	Offers Vitality Points for physical activity and health screenings, which can be used for gift cards and travel. Policyholders can save up to 15% on their life insurance by using internet-connected Fitbits

### Insurance Companies

In addition, we compiled a number of insurance companies that encourage the growth and uptake of wearable health technology through incentive programs: Oscar Health, United Healthcare, Humana, and John Hancock (see Table 2). These companies all offer health tracking through devices and promote the use of remote patient data to improve patient engagement and health. Key focus areas included patient data tracking and rewards programs for customers who use devices to track their health and achieve milestones. These rewards programs gamify health goals into point systems and offer incentives for customers, including gift cards, electronic devices, and travel. These initiatives by insurance companies support the uptake of wearable health technologies and expand the use of patient-collected data to improve patient health.

In addition to the tables above, we identified a number of health systems and organizations that engaged or stated interest in wearable health technology initiatives, such as NYU Langone Health, Penn Medicine, Duke Health, Novant Health, and Icahn School of Medicine at Mount Sinai. Such work includes integration of Fitbit and HealthKit data into patient health portals [60,76,77]. However, these groups have not yet published results from their work or current status of innovation. Limited reported information is likely to be because of the early stages of implementation and require follow-up in a future review of current innovations.

### Data Analysis

On the basis of the information collected from our survey sample of 10 start-up organizations and 4 insurance companies, the most common themes included a personalized patient experience based on health goals and past medical history, gamification through a rewards program, and data analytics capabilities (see Table 3).

We also recorded several key observations based on analyzed data:

1. Current rewards programs are strongly linked with wearable devices. Of the identified organizations, all rewards programs relied on the use of wearable devices to track data that could be used for patient incentives. The most common data point was step tracking; a patient could earn money or points to be traded in for prizes when they walked a certain number of steps each day.
2. AI capabilities are still limited. AI has yet to become fully established in the field of wearable health technology. A limited number of organizations are leveraging these digital capabilities to collect, analyze, and integrate patient data and monitoring and creating ongoing dialog about patient health activities.
3. There are varied approaches for personalization of patient information. Personalization of a patient’s experience was a prevalent theme across several of the surveyed organizations. The personalized experience was created through various approaches, including recommending health apps, facilitating ongoing conversations with a doctor or AI bot, or providing assessments so that a patient could better understand their health.
4. There are challenges and risks to all aspects of wearable health technology. Addressing system interoperability, patient privacy, and data overload risks will be critical to the use of wearable health technology. We mapped out the previously discussed challenges for each of the 6 themes in Table 4.

**Table 3:** Prevalence of wearable health technology themes across surveyed start-ups and insurance companies.

Theme	Number of surveyed organizations addressing themes
Personalized patient experience	4
Rewards program	4
Data analytics	3
Remote monitoring	2
Access to patient records	1
AI^a^ technology	1

^a^AI: artificial intelligence.

**Table 4:** Challenges and risks associated with wearable health technology.

Theme	Challenges		
	System interoperability	Patient privacy	Data overload
Personalized patient experience	—^a^	X^b^	—
Rewards program	—	X	—
Data analytics	—	—	X
Remote monitoring	X	X	X
Access to patient records	X	X	X
AI^c^ technology	—	X	—

^a^No expected challenge or risk associated with wearable technology theme.

^b^X: challenge or risk associated with wearable technology theme.

^c^AI: artificial intelligence.

## Discussion

### Principal Findings

This scoping study reviewed current innovations of wearable health technology and EHRs across health care systems, start-ups, and insurance companies and documented key innovation trends, partnerships, and incentives, along with challenges of wearables. Our findings reflect the movement toward the adoption of mobile health devices through the availability of digital tools and gamification of health data collection. However, numerous barriers to the efficient implementation of wearable health technology exist and are likely to hinder widespread adoption across health systems. Our report presents several current approaches to addressing wearable health technology and EHR integration barriers; these findings highlight the direction of wearable health innovation and serve to identify potential partnerships for future wearable adoption.

The development of technologies by start-ups outside of EHR systems highlights the interest in solving challenges in wearable health technology, such as information overload and system interoperability. Companies such as Redox are addressing interoperability issues by creating the technology to link hospitals’ EHR systems to outside applications regardless of software vendor. Others, such as Validic and Human API, are working to improve the workload for providers by simplifying the data collection from devices and outputting processed and easily understandable results.

Across the field of wearable health technology, maintaining patient privacy with the expanding use of wearables, rewards programs, remote monitoring, and AI continues to pose the greatest challenge to the growth of wearable health technology. Obtaining informed patient consent will be critical to provide clarity regarding what data are collected and which third parties can access patient data; this will continue to be a key discussion topic, as organizations seek to create a personalized patient experience based on patient-collected data. For example, companies such as Conversa allow for automated and personalized virtual care using conversational AI technology and patient remote data.

The implementation of health tracking rewards programs by insurance companies additionally signifies the interest and direction in which wearable health technology is moving to improve consumer health. These health insurance companies’ decisions to engage in wearables through rewards programs can offer increased opportunities in data collection and need for the above start-up technologies to provide a seamless experience for both providers and consumers. As wearable health technology becomes linked to gamification and rewards program initiatives for insurance companies, patient data integration across other platforms is also likely to become more commonplace.

This report serves as a starting point for those interested in wearable innovations rather than a comprehensive summary because of the rapidly changing nature of wearable health technology. As more institutions share their work in this area, address challenges, and create more efficient workflows/processes, the ability to transform patient care and streamline the integration of mobile health devices will improve the health outcomes and quality of care for patients.

### Limitations

Although we developed a detailed process to search and document the current state of wearables through our study, several challenges exist to create a comprehensive list. The nature of this report is Epic centric, as we were not able to access other internal EHR portals. There were a limited number of health systems actively publicizing or publishing their work on new integration methods through the general Web. Those that did also used different names (ie, remote data integration and device integration) that may not have been included in our search terms. Furthermore, based on the growing adoption of wearable health technology in health systems over the past few years, we anticipate that new names would have been added to this list since our search.

### Conclusions

Wearable health technology will play a critical role in greater transparency between patients and providers and chronic condition management. Devices and technologies that enable the streamlined movement of data from patients to providers are key to improving a patient’s care journey and empowering them to manage their own health. The future design and development of digital technology in this space will rely on continued analysis of best practices, pain points, and potential solutions to mitigate existing challenges.

By sharing our results, we have presented key challenges and emerging solutions to this rapidly evolving field. Our work serves as an initial foundation to the creation of a streamlined process to identify relevant entities in this field and provide resources on the implementation of and workflows around wearable health technology and EHR integration for organizations across the health care industry. As much of this work is still ongoing, we anticipate that these findings will serve as the foundation for future studies on wearable health technology.

## Abbreviations

AI: artificial intelligence
API: application programming interface
EHR: electronic health record
HIPAA: Health Insurance Portability and Accountability Act of 1996
IT: information technology
UI: user interface
